# Developing a population-scale harmonised ethnicity-spine in Wales.

**DOI:** 10.23889/ijpds.v7i3.1930

**Published:** 2022-08-25

**Authors:** Ashley Akbari, Stuart Bedston, Hoda Abbasizanjani, Gareth Davies, Richard Fry, Emily Lowthian, Jane Lyons, Rhiannon Owen, Fatemeh Torabi, Kamlesh Khunti, Ronan Lyons

**Affiliations:** 1 Population Data Science, Swansea University; 2 Diabetes Research Centre, University of Leicester

The COVID-19 pandemic has placed a spotlight on existing and enduring health inequalities experienced by different ethnic groups. There has been a longstanding call to generate and improve the use of ethnicity data available across different data sources, in order to improve our understanding of health risks, behaviours and outcomes.

We used multiple anonymised individual-level population-scale data sources available within the Secure Anonymised Information Linkage (SAIL) Databank to develop a harmonised ethnicity spine for the population of Wales. We documented ethnicity information in multiple longitudinal records from January 2000 onwards. Data sources included: health and social care, birth and mortality records, national census records, specialist clinical audits and registers, surveys and other routine electronic data. To enable multi-source harmonisation, we explored the ethnicity categorisation as well as temporal changes in recording and classifications by obtaining distribution of records for population, which informed our harmonisation algorithm for standardisation of ethnicity records.

We used over 20-data sources on ~5-million individuals, spanning varying time-periods starting from January 2000 upto a maximum of 22-years. We harmonised available recorded ethnicity values into standardised ethnic classification groups within a national ethnicity-spine. Furthermore, we investigated the impact of different harmonisation methods, including composite, latest date of recording, modal and weighted modal results. With the main focus of the methodological development being in response to the COVID-19 pandemic, when linked to the ~3.1 million individuals alive and resident in Wales from January 2020, we generated harmonised ethnic groups towards ~95% completeness in data coverage for the whole population of Wales. The predominant ethnic group in Wales observed was White, accounting for 89% of the population when using the latest date of recording method.

This research highlights challenges in using longitudinal ethnicity data across different sources. Further work is needed to understand the basis on which individuals / organisations record ethnicity overtime. We recommend improvements recognising differences between ethnicity and other social constructs (e.g. ancestry, nationality, country of origin) are better documented / understood.

